# Prognostic significance of circulating tumour cells following surgical resection of colorectal cancers: a systematic review

**DOI:** 10.1038/sj.bjc.6605651

**Published:** 2010-04-13

**Authors:** G Peach, C Kim, E Zacharakis, S Purkayastha, P Ziprin

**Affiliations:** 1 Division of Surgery, Department of Surgery and Cancer, Faculty of Medicine, St Mary's Campus, Imperial College London, Praed Street, London W2 1NY, UK; 2 Imperial College School of Medicine, Imperial College London, London SW7 2AZ, UK

**Keywords:** colorectal cancer, circulating tumour cells, prognosis, recurrence, postoperative

## Abstract

**Background:**

The role of adjuvant chemotherapy after resection of colorectal cancers (CRCs) is well understood for patients with stage-I or stage-III disease. Its efficacy for those with stage-II disease remains much less clear. Many investigators have sought to identify prognostic markers that might clarify which patients have the highest risk of recurrence and would, therefore, be most likely to benefit from chemotherapy. This systematic review examines evidence for the use of peripherally sampled, circulating tumour cells (CTCs) as such a prognostic marker.

**Methods:**

A comprehensive literature search was used to identify studies reporting on the significance of CTCs in the postoperative blood of CRC patients.

**Results:**

Fourteen studies satisfied the inclusion criteria. Six of the nine studies that took blood samples 24 h or more postoperatively found detection of postoperative CTCs to be an independent predictor of cancer recurrence.

**Conclusion:**

The presence of CTCs in peripheral blood at least 24 h after resection of CRCs is an independent prognostic marker of recurrence. Further studies are needed to clarify the optimal time point for blood sampling and determine the benefit of chemotherapy in CTC-positive patients with stage-II disease.

In modern clinical practice, the accepted treatment for non-metastatic colorectal cancer (CRC) is tumour resection, with or without adjuvant chemotherapy depending on tumour stage. For stage-I cancers, surgical resection is generally curative without the need for chemotherapy, whereas for stage-III (node-positive) cancers, adjuvant chemotherapy has been shown to significantly improve outcomes (1990). For patients with stage-II (node-negative) cancers, however, the benefits of adjuvant chemotherapy remain much less clear. For these patients, any potential benefits are often outweighed by the risks and side-effects of treatment (Benson *et al*, 2004). For this reason, the international QUASAR trial was set up to assess the efficacy of adjuvant chemotherapy in this patient group. QUASAR concluded that although adjuvant chemotherapy may improve survival of patients with stage-II CRC, the absolute improvement in 5-year survival was minimal at only 3.6% (Quasar Collaborative Group, 2007).

With recurrence rates (RRs) being highly variable for patients with stage-II disease, investigators have sought to differentiate between those at high risk and those at low risk of recurrence. By distinguishing these two subgroups it might be possible to identify those patients most likely to benefit from adjuvant chemotherapy and target treatment accordingly. Previously established prognostic markers for cancers at high risk of recurrence include local tumour extent, regional lymph node metastasis, blood or lymphatic vessel invasion, residual tumour after curative surgery, and preoperative elevation of carcinoembryonic antigen (CEA) (Compton *et al*, 2000). More recently, molecular detection of circulating tumour cells (CTCs) has also been investigated as a potential prognostic marker.

Although previous analyses have demonstrated some prognostic significance for CTC levels in portal blood sampled intraoperatively (Katsuno *et al*, 2008), studies assessing the significance of preoperative CTC levels have shown little predictive value in terms of clinical outcome (Wharton *et al*, 1999; Tsavellas *et al*, 2001). Other reviews have also assessed the significance of CTC levels, but these have included relatively few studies with information on postoperative CTC detection (Sergeant *et al*, 2008), and early findings have suggested that this postoperative detection of CTCs may actually offer greatest prognostic value. We therefore undertook a systematic review of the evidence for the use of peri- and postoperative CTC detection in predicting the outcome following surgical resection of colorectal cancers (CRCs).

The objectives of this systematic review were (1) to examine current literature and clarify the prognostic significance of peripherally sampled CTCs after resection of non-metastatic CRCs; (2) to identify those markers most likely to be of use in risk-stratification of CRC patients after potentially curative surgery; and (3) to identify the most appropriate directions for further research.

## Materials and methods

The measurement tool for ‘assessment of multiple systematic reviews’ (AMSTAR) was used as a reference. This tool consists of 11 items and has good face and content validity for measuring the methodological quality of systematic reviews (Shea *et al*, 2007).

### Literature search

A literature search was performed using multiple electronic search engines, including Medline (using PubMed), the Cochrane Database, Ovid, and Google Scholar. Studies reporting on the molecular detection of CTCs in postoperative peripheral blood and its effect on prognosis in CRC (last search date 1st December 2009) were identified. The following keywords were used for the search: ‘colon’, ‘rectal’, ‘colorectal cancer’, ‘circulating cells’, ‘prognosis’, ‘mRNA’, ‘PCR’, ‘-breast’, ‘-gastric’. The ‘related articles’ function in PubMed and Google Scholar was also used to identify additional articles. References of the articles identified were also searched for by title and then subsequent abstract review.

### Eligibility criteria and data extraction

All published studies reporting on the effect of CTCs in postoperative peripheral blood in CRC on prognosis were considered. There were no restrictions made on the type of study.

Data were extracted on author, year of publication, study design, technical aspects of the studies, and outcomes. All data were extracted by two reviewers (C Kim and G Peach), and any discrepancies were resolved by consensus from all authors. Restrictions were made to papers published in the English language.

### Inclusion and exclusion criteria

To be included in the analysis, studies had to (1) include patients undergoing curative resection of CRC; (2) include peri- or postoperative CTC detection; (3) report on at least one of the outcome measures listed in the next section; and (4) include peripheral blood samples. When two studies were reported by the same institution and/or authors, both were included in the analysis.

Studies were excluded from the analysis if (1) outcomes of interest were not reported; (2) it was impossible to extract or calculate the necessary data from the published results; (3) it only reported on the preoperative sampling of peripheral blood; and (4) only portal and/or mesenteric blood sampling was undertaken.

### Outcomes of interest and definitions

The outcomes of interest were any type of prognostic data, including: disease-free survival (DFS), overall survival (OS), RR, cancer-specific survival, and odds and hazards ratios (HRs).

### Data analysis

Raw data on outcomes of interest were collected and tabulated. The data were stored on Microsoft Office Excel.

## Results

**Figure 1:**
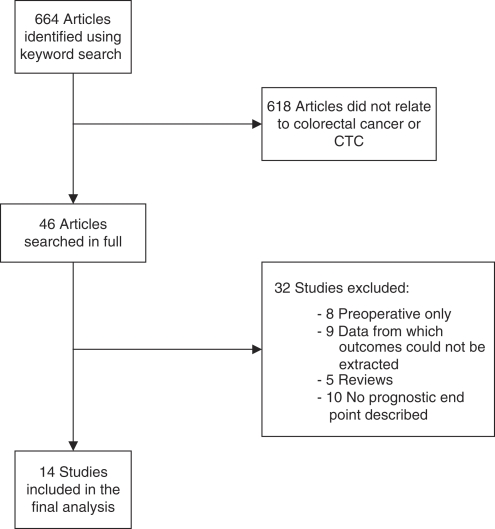
Flow diagram describing the selection of studies included in this review.

Six hundred and sixty-four articles were identified using the above keywords. Title and abstract review resulted in the exclusion of 618 articles that did not address the molecular detection of CTCs and prognosis in CRC. Forty-six references were assessed in full, and a further 32 studies were excluded (Figure 1). Eight studies were excluded as they reported only on preoperative blood sampling (Bessa *et al*, 2001; Fujita *et al*, 2001; Wong *et al*, 2001; Zhang *et al*, 2005; Douard *et al*, 2006; Iinuma *et al*, 2006; Wang *et al*, 2006; Friederichs *et al*, 2007). Nine studies were excluded as they contained data from which outcomes could not be extracted, of which four were excluded as they combined positive results of molecular detection of multiple samples and their effect on prognosis (Hardingham *et al*, 2000; Guller *et al*, 2002; Bosch *et al*, 2003; Koyanagi *et al*, 2008), and five were excluded as it was impossible to extract the data for outcomes of interest (White and Griffiths, 1976; Funaki *et al*, 1998; Wyld *et al*, 1998; Conzelmann *et al*, 2005; Wang *et al*, 2007). Five studies were excluded as they were reviews or systematic reviews, which did not report on prognostic outcomes (Tsavellas *et al*, 2001; Sleijfer *et al*, 2007; Riethdorf *et al*, 2008; Sergeant *et al*, 2008; Tsouma *et al*, 2008). Ten studies were excluded as they did not report on prognostic outcomes (Denis *et al*, 1997; Nakamori *et al*, 1997; Douard *et al*, 2001; Guadagni *et al*, 2001; Patel *et al*, 2002; Silva *et al*, 2002; Yokoyama and Yamaue, 2002; Schuster *et al*, 2004; Yeh *et al*, 2006; Lagoudianakis *et al*, 2009). The outcomes measured in the 14 studies included in this review were too heterogeneous with regards to the methodology of data collection to allow quantitative analysis. A systematic review was therefore undertaken.

### Study characteristics

The 14 studies included here comprised 1841 patients in total, with the median number of patients in each study being 99.5 (range 42–438) (Taniguchi *et al*, 2000; Yamaguchi *et al*, 2000; Ito *et al*, 2002; Bessa *et al*, 2003; Chen *et al*, 2004; Sadahiro *et al*, 2005, 2007; Katsumata *et al*, 2006; Koch *et al*, 2006; Lloyd *et al*, 2006; Allen-Mersh *et al*, 2007; Uen *et al*, 2007, 2008; Wang *et al*, 2007).

Of the 14 studies included, eight undertook blood sampling peri-operatively (Taniguchi *et al*, 2000; Yamaguchi *et al*, 2000; Ito *et al*, 2002; Chen *et al*, 2004; Sadahiro *et al*, 2005; Katsumata *et al*, 2006; Koch *et al*, 2006; Lloyd *et al*, 2006), four undertook sampling approximately 24 h after resection (Bessa *et al*, 2003; Koch *et al*, 2006; Lloyd *et al*, 2006; Allen-Mersh *et al*, 2007), and six undertook sampling between 24 h and 14 days after resection (Chen *et al*, 2004; Allen-Mersh *et al*, 2007; Sadahiro *et al*, 2007; Uen *et al*, 2007, 2008; Wang *et al*, 2007).

### Tumour stage

The studies examined in this review included patients with various different stages of disease. Seven investigated the role of CTC in patients with stage-I to stage-III disease undergoing potentially curative surgery (Ito *et al*, 2002; Bessa *et al*, 2003; Sadahiro *et al*, 2005, 2007; Allen-Mersh *et al*, 2007; Wang *et al*, 2007; Uen *et al*, 2008); three studied CTC in patients with early CRC (of which two investigated stage-II disease alone (Koch *et al*, 2006; Uen *et al*, 2007) and one investigated stage-I and stage-II disease (Lloyd *et al*, 2006)); and four papers included patients with stage-I to stage-IV disease (Taniguchi *et al*, 2000; Yamaguchi *et al*, 2000; Chen *et al*, 2004; Katsumata *et al*, 2006).

### Blood sampling site

Of the 14 studies included in our analysis, 10 involved sampling of peripheral blood only (Ito *et al*, 2002; Bessa *et al*, 2003; Chen *et al*, 2004; Katsumata *et al*, 2006; Lloyd *et al*, 2006; Allen-Mersh *et al*, 2007; Sadahiro *et al*, 2007; Uen *et al*, 2007, 2008; Wang *et al*, 2007); 1 used peripheral and portal blood samples (Sadahiro *et al*, 2005); 1 used peripheral and mesenteric samples (Yamaguchi *et al*, 2000); 1 used peripheral, portal and mesenteric samples (Taniguchi *et al*, 2000); and 1 used samples of central venous blood (Koch *et al*, 2006).

### CTC detection methods

Thirteen of the 14 included studies used identification of specific mRNA to detect the presence of CTCs. Eleven of these used reverse transcriptase-PCR (RT-PCR) to detect tumour cells in the sampled blood (Taniguchi *et al*, 2000; Yamaguchi *et al*, 2000; Ito *et al*, 2002; Bessa *et al*, 2003; Chen *et al*, 2004; Sadahiro *et al*, 2005, 2007; Katsumata *et al*, 2006; Koch *et al*, 2006; Allen-Mersh *et al*, 2007; Uen *et al*, 2007) whereas the other two, both from the same unit, used a membrane array (Wang *et al*, 2007; Uen *et al*, 2008). The remaining study used immunocytochemical staining of circulating cells after immunomagnetic purification (Lloyd *et al*, 2006).

Seven of the included studies used a single marker to detect CTCs, with the chosen marker being CEA mRNA (*n*=5), cytokeratin-20 (CK20), or guanylyl cyclase (GCC) (Taniguchi *et al*, 2000; Ito *et al*, 2002; Bessa *et al*, 2003; Chen *et al*, 2004; Sadahiro *et al*, 2005, 2007; Koch *et al*, 2006). The other studies used multiple markers, with an overall CTC-positive result usually being determined by detection of more than one marker.

### CTC detection rates

There was a mean detection rate of 33.4% (±3.6 s.e.m.). There were no differences between studies that sampled peri-operatively, early postoperatively, or late postoperatively, nor were there any differences between studies that included only early-stage disease, curative patients only, and patients at all stages. Furthermore, there was no demonstrable difference in detection rate between studies that used one, two, or multiple markers. Direct comparison between the different cellular markers could not be made due to differences in methodology.

In the three series that only investigated patients with early-stage disease (i.e., stage-I and/or stage-II) the detection rates were variable. Koch *et al* (2006) and Uen *et al* (2007) reported positivity rates of 34 and 27%, respectively, whereas the paper by Lloyd *et al* (2006) only reported a positive detection rate of less than 5% in the peripheral blood postoperatively. This paper described the use of an immunobead technique to purify blood cells, which may account for any differences.

### Patient follow-up

Median follow-up (where stated) ranged from 36 to 68 months. In the 10 papers that clearly stated the length of follow-up, there was no identifiable relationship between length of follow-up and the reported level of CTC significance.

### Prognostic influence of CTCs

#### Peri-operative sampling

In the first of the studies to take peri-operative samples (Table 1), Taniguchi *et al* (2000) took samples of both peripheral and portal blood intra-operatively (before mobilisation of the tumour) and examined them for CEA mRNA using RT-PCR. Thirty-four percent of patients showed CTC positivity in peripheral blood and after potentially curative surgery; this group of patients had a lower DFS (50 *vs* 74% at 2 years; *P*<0.01) than patients without detectable CTC. Conversely, although Sadahiro *et al* (2005) also took peripheral blood samples intra-operatively (pre-resection) and also used a CEA mRNA protocol, they failed to show any link between CTC positivity and tumour recurrence or survival. Ito *et al* also tested for CEA mRNA using RT-PCR, but took samples immediately after operation. They showed positivity rates of 37% and showed that those patients who were CTC-positive once again had reduced DFS (77 *vs* 92% at 58 months; *P*=0.0296).

All other studies that took peri-operative samples used detection of multiple cellular markers. Yamaguchi *et al* (2000) showed that positivity for both CEA and CK20 in blood samples obtained intra-operatively was associated with a trend towards decreased survival (60 *vs* 85% at 1 year), although this was not a statistically significant difference (*P*=0.06). Katsumata *et al* (2006) chose to use CEA, CK19, and CK20 mRNA as markers to detect CTCs in patients with stage-I to stage-IV disease undergoing surgery. They found both CK19 and CEA positivity to be unrelated to tumour progression. However, they also showed that whereas CK20 expression had no correlation with local tumour recurrence, it did correlate with the presence of lymph node metastasis (*P*=0.037), and that 5-year survival in the CK20-positive group was 65.2 *vs* 87.5% in the CK-negative group (*P*=0.048). Nonetheless, CTC positivity as determined by CK20 was not found to be an independent prognostic marker. This apparent discrepancy is probably related to CTC positivity reflecting stage (as shown by its correlation with lymph node metastases) rather than prognosis.

Lloyd *et al* (2006) used CK20, CEA ephrin-B4, laminin-*γ*2, and matrilysin mRNA as markers of CTC in peripheral samples taken preoperatively and intra-operatively (as well as post-operatively – see below). They did not show any significant correlation between CTC positivity and survival although mean survival was only 36.5 months in CTC-positive patients as compared with 76 months in negative patients. It should be noted that although patients in this study had early disease (stage-I and stage-II only), the number to test positive for any marker postoperatively was very low (4%) compared with other papers in this review. Furthermore, none of the patients were positive for circulating CEA mRNA preoperatively, in contrast to other studies discussed here.

Finally, Chen *et al* (2004) used GCC as a marker of CTCs in peripheral blood samples taken pre-, intra-, and postoperatively. Whereas CTC positivity in preoperative and intra-operative samples did not correlate with prognosis, patients who were CTC positive in samples taken 14 days postoperatively had significantly lower DFS and OS (see below).

#### Early postoperative sampling (0–48 h)

In the first study that looked at postoperative sampling (Table 2), Bessa *et al* (2003) investigated 66 patients with stage-I to stage-III disease undergoing potentially curative surgery. They used RT-PCR to detect CEA mRNA in peripheral blood both before and 24 h after surgery. No data were presented in relation to the prognostic significance of preoperative CTC positivity. Postoperative samples showed a 55% positivity rate but RR were almost identical in both CEA mRNA-positive and negative groups (22 *vs* 23%, respectively, at 36 months). Similarly, postoperative CTC positivity did not influence OS. After adjusting for TNM stage, the probability of RRs and OS rates were also the same in both groups. In addition to taking preoperative and intra-operative samples (see above), Lloyd *et al* (2006) also took peripheral blood samples the morning after surgery. Unlike Bessa *et al*, they found that mean survival was reduced in patients who were CTC-positive at this time point, but this was not a statistically significant result (mean survival 42.6 months *vs* 75.9; *P*=0.6). However, it is interesting to note that in this study the postoperative positivity rate was very low (4.2%), just as it had been in their preoperative samples – even though high positivity rates might be expected as positivity was defined as presence of any one of the five markers used.

Allen-Mersh *et al* (2007) took peripheral samples and used RT-PCR to detect both CK20 and CEA. Samples were taken preoperatively, 24 h after resection, and at 1 week after operation (see below). They found no significant difference in outcome between patients who were CTC-positive preoperatively (as defined by presence of either marker) and those who were CTC-negative preoperatively. However, patients who were CTC-positive 24 h postoperatively had much poorer DFS (56 *vs* 90% at 2 years; *P*<0.001). These results are supported by the findings of Koch *et al* (2006) who took samples of central venous blood at 24 h after resection (as well as peri-operatively – see above). This group investigated only patients who had stage-II disease and CK20 was used as the detection marker. They showed that CTC positivity at 24 h was associated with reduced 5-year relapse-free survival (70 *vs* 93% *P*=0.003) and disease-specific survival (77 *vs* 100% *P*=0.0006), the latter regardless of whether patients had received chemotherapy or not. Furthermore, multivariate analysis confirmed that identification of CTC tumour cell in the peripheral blood at 24 h after surgery was an independent predictor of relapse and disease-specific mortality (HR 2.4 (1.3–5.3); *P*=0.008, and HR 6.4 (2.2–34); *P*=0.0003, respectively).

### Late postoperative sampling (>48 h)

A number of studies have also looked at the role of CTCs persisting more than 48 h after surgery (Table 3). Sadahiro *et al* (2007) used RT-PCR to detect CEA in peripheral blood samples taken 7–10 days after resection from patients with stage-I to stage-III disease. The overall rate of postoperative CTC positivity was 22% and CTC-positive patients showed significantly poorer DFS (51 *vs* 70% at 5 years; *P*=0.007) and OS (65 *vs* 80% at 5 years; *P*=0.04) than CTC-negative patients. They also found that even among patients with stage-I disease, RRs were much higher in the CEA mRNA-positive group (45 *vs* 22% *P*=0.003). Overall, they were able to show that CEA mRNA positivity was a significant independent risk factor for tumour recurrence (RR 2.29 (1.30–4.02)) but not for OS (RR 1.81 (0.94–3.50)).

Three studies by the same group (Uen *et al*, 2007, 2008; Wang *et al*, 2007) used a membrane array technique to detect multiple cellular markers. These were CK20, CK19, CEA, and human telomerase reverse transcriptase (hTERT), and CTC positivity was defined as presence of all four of these markers. In 194 patients with stage-II disease undergoing curative surgery, 27.5% were positive for CTC at least one week after surgery. Almost 85% of positive patients relapsed whereas only 7.8% of patients negative for CTC developed recurrence (Uen *et al*, 2007). Positivity was shown to be a predictor of relapse (HR 38.6, 13.9–106.9), greater than T-stage or vascular invasion alone. Similarly, when looking at all patients undergoing curative surgery (i.e., those with stage-I to stage-III disease), the same authors again showed that CTC positivity at 1 week after resection was a prognostic indicator for recurrence (*P*<0.001; HR 29.5 (10.3–87.8)). Worse DFS was also seen in patients with CTC detected postoperatively (*P*<0.001). This group also looked at the prognostic effect of CTC detected 4 weeks after surgery using the same membrane array for the described markers and again showed significant association between positive CTC and recurrence (HR 18.7 (5.6–112.8)).

Although Chen *et al* (2004) were unable to show any prognostic significance of preoperative and intra-operative CTC levels (see above), they too found late postoperative CTC levels to be a highly significant indicator of outcome. At 14 days after resection, patients who were CTC-positive (defined as more than 10^2^ CTC per 10^6^ nucleated blood cells) had much worse DFS (50 *vs* 94% at 36 months; *P*=0.001) and OS (65 *vs* 94% at 36 months; *P*=0.039) than those who were CTC-negative. No multivariate analysis was performed to investigate whether high CTC load was an independent risk factor.

Allen-Mersh *et al* (2007) not only took samples preoperatively and at 24 h after operation (see above), but also at 1 week after resection. At this time point they were unable to show any difference in prognosis between the CTC-positive and CTC-negative groups, although this is most probably due to the fact that there were very few positive results by 1 week after the operation and this would render any differences between the two groups statistically insignificant.

## Discussion

Staging of disease with Dukes' or TNM systems is generally used to help predict recurrence and cancer-specific survival, and identify patients who may benefit from adjuvant chemotherapy. As indiscriminate use of chemotherapy for patients with stage-II disease results in minimal reduction in mortality, identification of a new prognostic marker may help to determine which patients would benefit most. Although the majority of the studies included in this review did not limit their investigations to patients with stage-II disease, their findings may provide good basis for larger subsequent trials in this patient group.

The papers presented here did not show peri-operative CTC levels to be of value in predicting recurrence of CRC. This echoes the findings of most previous studies looking at preoperative markers, and whereas Koyanagi *et al* (2008) found preoperative detection of CTCs to be an independent prognostic indicator, this has not been supported by earlier reviews (Wharton *et al*, 1999; Tsavellas *et al*, 2001). Some also noted differences in DFS between those who were CTC-positive preoperatively and those who were CTC-negative. This may simply be a reflection of tumour stage however, as several of these papers found CTC detection rate to be dependent on other clinicopathological characteristics (such as T-stage and lymph node status) that are known to have prognostic significance.

Six of the nine papers investigating the prognostic role of CTCs persisting 24 h or more after resection found CTC detection to be an independent predictor of cancer recurrence (Koch *et al*, 2006; Allen-Mersh *et al*, 2007; Sadahiro *et al*, 2007; Uen *et al*, 2007, 2008; Wang *et al*, 2007). Furthermore, 2 out of 4 papers using early postoperative sampling showed that CTC detected as early as 24 h postoperatively had significant influence on recurrence (Koch *et al*, 2006; Allen-Mersh *et al*, 2007). Koch *et al* (2006) also showed that detection of CTC predicted poorer cancer specific survival. Unlike Koch *et al*, Lloyd *et al* (2006) did not show any relationship between CTC detection and cancer-specific outcomes. However, it is interesting to note that although patients in this study had early disease (stage-I and stage-II only), positivity rate for any of the five markers used was very low at 4.2% and only 1 out of 113 patients was positive for circulating CEA mRNA postoperatively. This is in stark contrast to the other studies, which showed positivity rates between 22 and 62.9%. Even the studies by Koch *et al* (2006) and Uen *et al* (2007) (which included stage-II patients only) had positivity rates of 34 and 27.5%, respectively. This may be explained by the unique immunomagnetic bead methodology that Lloyd *et al* used to isolate cells from the blood. With such low rates of CTC detection, it is highly unlikely that they would have been able to show a significant association between persisting CTC and recurrence.

Bessa *et al* (2003) also failed to show any association between CTC positivity at 24 h and recurrence. However, it is once again interesting to note that in this study all 28 patients with stage-II disease received 5-fluorouracil-based adjuvant chemotherapy, unlike in the studies by Koch *et al* (2006) and Allen-Mersh *et al* (2007), where very few node-negative patients received chemotherapy (6 and 13% of patients, respectively). This use of chemotherapy for patients studied by Bessa *et al* may well have improved the outcomes of CTC-positive patients with stage-II disease, thereby masking any potential difference between the two groups. Furthermore, false-positive and false-negative rates were not presented in this study and may have influenced their results.

The presence of free CTC is dependent on two processes: dissemination of cancer cells into the circulation and subsequent elimination of those cells. Dissemination is thought to occur from both the primary tumour site and from occult micrometastases, such as bone marrow, lung, or liver (Pantel and Brakenhoff, 2004). Surgical manipulation may increase CTC release from the primary tumour during surgery and make peri- or early postoperative sampling unreliable. This could explain why peri-operative sampling has not been shown to be of prognostic significance (as found in this review) and why few previous studies have been able to show a role for preoperative CTC. In terms of dissemination from micrometastases, ongoing release of CTC from these sites may explain why some patients have persistently high CTC levels even after resection. This might contribute to the apparent association between high postoperative CTC levels and poor prognosis, as high CTC levels could signify more systemically advanced disease.

Elimination of CTC may also occur by a number of pathways, including anoikis, mechanical stress, microembolie (Geiger and Peeper, 2009), and possibly immunological eradication (Johansson *et al*, 2008). Although it has previously been suggested that most CTC are cleared from the circulation within 24 h of tumour resection (Fidler, 1970; Patel *et al*, 2002), persisting CTCs may have increased resistance to these degradation processes and therefore show greater metastatic potential (Song *et al*, 2001). This would also be in keeping with the demonstrated correlation between long-surviving tumour cells and poor prognosis. The significance of rate of CTC clearance was not generally assessed in detail in the papers analysed here. Nonetheless, the demonstrated correlation between persisting CTC positivity and poor prognosis may suggest that more rapid clearance would be associated with better prognosis. This is supported in a previous study by Patel *et al* (2002), which found that clearance of circulating tumour cells within 24 h of CRC excision was greatest in patients with stage-I and stage-II disease.

For CTC testing to be used clinically as a prognostic indicator, it is also important to establish the most reliable time point for blood sampling. The evidence presented here suggests that when testing is undertaken at least 24 h after surgery, presence of CTC has a prognostic role: Allen-Mersh *et al* (2007) and Koch *et al* (2006) both showed significance at 24 h, with an additional five papers showing a clear role for testing between 7 and 14 days postoperatively. However, as direct comparison between these time points has not yet been undertaken, the optimal timing for collection of blood samples has yet to be clarified.

Whereas use of multiple cellular markers might be expected to increase sensitivity and specificity, detection rates were found to be similar in each series and across all markers. The studies from Taiwan (Chen *et al*, 2004; Uen *et al*, 2007, 2008; Wang *et al*, 2007) used an RNA array to detect four different markers, whereas others showed a prognostic role for CTC using either CEA or CK20 mRNA (or both). A number of additional factors may also affect the results. These include such things as use of chemotherapy and differences in RT-PCR methodology, which may vary due to the method of blood preparation or the time lapse between sampling and processing. These are not examined in more detail here, as meta-analysis was not possible due to heterogeneity of existing data. One might also expect variations in study size to affect the degree to which CTC significance could be shown. However, whereas the more highly powered studies did indeed tend to show greatest significance, studies with relatively few patients (e.g., Koch *et al*, 2006) were also able to show prognostic significance of CTC.

## Conclusion

The presence of CTCs in peripheral blood at least 24 h after resection of CRCs is an independent prognostic marker of recurrence. However, further studies are needed to clarify the optimal time point for postoperative blood sampling, and to identify the most reliable cellular marker for measurement of CTC level. The evidence presented here provides a possible basis for future large-scale, multi-centre trials with more unified methodology. By including a greater number of patients with stage-II disease, it may be possible to clarify the significance of CTC within this subgroup and more accurately identify those patients most likely to benefit from chemotherapy.

## Figures and Tables

**Table 1:** Characteristics of studies that took blood samples peri-operatively

											**Outcome result**		
**First author**	**Year**	**No. of pts**	**Median age (years)**	**Dukes/TNM stage**	**Blood sampling site**	**Time sample taken**	**Cellular marker used**	**Assay method**	**Positivity rate (%)**	**Outcome measured**	**CTC +ve**	**CTC −ve**	**Hazard ratio (95% CI)**
Chen	2004	42	64.2	1–4	P	3 day preop, peri-op, 14 days post	GCC	R	—	DFS at 36 months	No correl	No correl	Not stated
Ito	2002	99	—	1–3	P	Immed preop, immed postop	CEA	R	50.5	DFS at 58 months	77%	92%	Not stated
Katsumata	2006	57	66.1	A, B, C, D	P	Intra-op	CEA CK19 CK20	R	42.1	DFS at 5 years	65.2%	87.5%	1.63 (CK20) 0.24 (CEA) (CI not stated)
Koch	2006	90	66	2	CV	Peri-op, 24 h post	CK20	R	24	DFS at 5 years	72%	90%	Not stated
Lloyd	2006	146	74	1, 2	P	Preop, intra-op, 24 h post	CEA/LAMg2/CK-20/EphB4/MAT	R	4	Mean survival	36.5%	76%	Not stated
Sadahiro	2005	100	—	1–3	P, Po	Intra-op	CEA	R	37	DFS	No diff	No diff	0.24 (0.05, 1.12)
Taniguchi	2000	53	65	A, B, C, D	P, Po, mes	Intra-op	CEA	R	34	DFS at 2 years	50%	74%	Not stated
Yamaguchi	2000	52	—	1–4	P, mes	Preop, intra-op	CEA CK20	R	44.4	1-year survival	60%	85%	3.38 (periph CEA+CK20) (*P*=0.353)

Abbreviations: CEA=carcinoembryonic antigen; CI=confidence interval; CK19=cytokeratin-19; CK20=cytokeratin-20; CTC=circulating tumour cell; CV=central venous; DFS=disease-free survival; GCC=guanylyl cyclase-C; hTERT=human telomerase reverse transciptase; LAMg2=laminin-5g2; MA=membrane array; MAT=matrilysin; mes=mesenteric; OS=overall survival; P=peripheral; Po=portal; R=reverse-transcriptase-PCR.

**Table 2:** Characteristics of studies that took blood samples 24 h after resection

											**Outcome result**		
**First author**	**Year**	**No. of pts**	**Median age (years)**	**Dukes/TNM stage**	**Blood sampling site**	**Time sample taken**	**Cellular marker used**	**Assay method**	**Positivity rate (%)**	**Outcome measured**	**CTC +ve**	**CTC −ve**	**Hazard ratio (95% CI)**
Allen-Mersh	2007	147	67.4	A, B, C	P	Preop, 24 h post, 1 week post	CEA CK20	R	31	DFS at 2 years	56%	90%	8.6 (3.0, 24.3)
Bessa	2003	66	73	1–3	P	Preop, 24 h post	CEA	R	55	Recurrence at 36 months	22%	23%	1.54 (0.4, 5.0)
Koch	2006	90	66	2	CV	Peri-op, 24 h post	CK20	R	34	DFS at 5 years	70%	93%	2.4 (1.3, 5.3)
Lloyd	2006	146	74	1, 2	P	Preop, intra-op, 24 h post	CEA/LAMg2/CK-20/EphB4/MAT	R	4	Mean survival	42.6 m	75.9 m	Not stated

Abbreviations: CEA=carcinoembryonic antigen; CI=confidence interval; CK19=cytokeratin-19; CK20=cytokeratin-20; CTC=circulating tumour cell; CV=central venous; DFS=disease free survival; GCC=guanylyl cyclase-C; hTERT=human telomerase reverse transciptase; MA=membrane array; MAT=matrilysin; LAMg2=laminin-5g2; mes=mesenteric; OS=overall survival; P=peripheral; Po=portal; R=reverse-transcriptase-PCR.

**Table 3:** Characteristics of studies that took blood samples >48 h after resection

											**Outcome result**		
**First author**	**Year**	**No. of pts**	**Median age (years)**	**Dukes/TNM stage**	**Blood sampling site**	**Time sample taken**	**Cellular marker used**	**Assay method**	**Positivity rate (%)**	**Outcome measured**	**CTC +ve**	**CTC −ve**	**Hazard ratio (95% CI)**
Allen-Mersh	2007	147	67.4	A,B,C	P	Preop, 24 h post, 1 week post	CEA CK20	R	40.7	DFS at 2 years	No diff	No diff	2.91 (1.08, 7.85)
Chen	2004	42	64.2	1–4	P	3 day preop, peri-op, 14 days post	GCC	R	28.6	DFS and (OS) at 36 months	50% (65%)	94% (94%)	Not stated
Sadahiro	2007	200	59	1–3	P	7–10 days postop	CEA	R	22	DFS and (OS) at 5 years	50% (65%)	94% (94%)	2.29 (1.3, 4.0)
Uen	2007	194	64.9	2	P	>1 week postop	hTERT CK19 CK20 CEA	R	27.5	Recurrence at 40 months	85%	8%	38.6 (all 4 markers) (13.9, 106.9)
Uen	2008	438	65.6	1–3	P	1 days preop, 7 days postop	hTERT CK19 CK20 CEA	MA	31.3	DFS at 5 years	46%	86%	29.5 (all 4 markers) (10.3, 87.8)
Wang	2007	157	65.8	1–3	P	14 days postop	CK19 CK20 CEA hTERT	MA	57.3	Recurrence at 36 months	50%	80%	18.5 (all 4 markers) (5.6, 112.8)

Abbreviations: CEA=carcinoembryonic antigen; CI=confidence interval; CK19=cytokeratin-19; CK20=cytokeratin-20; CTC=circulating tumour cell; CV=central venous; DFS=disease-free survival; GCC=guanylyl cyclase-C; hTERT=human telomerase reverse transciptase; LAMg2=laminin-5g2; MA=membrane array; MAT=matrilysin; mes=mesenteric; OS=overall survival; P=peripheral; Po=portal; R=reverse-transcriptase-PCR.
